# Sex Differences in the Rapid Detection of Emotional Facial Expressions

**DOI:** 10.1371/journal.pone.0094747

**Published:** 2014-04-11

**Authors:** Reiko Sawada, Wataru Sato, Takanori Kochiyama, Shota Uono, Yasutaka Kubota, Sayaka Yoshimura, Motomi Toichi

**Affiliations:** 1 Primate Research Institute, Kyoto University, Inuyama, Aichi, Japan; 2 Faculty of Human Health Science, Graduate School of Medicine, Kyoto University, Kyoto, Japan; 3 Health and Medical Services Center, Shiga University, Otsu, Shiga, Japan; University of Groningen, Netherlands

## Abstract

**Background:**

Previous studies have shown that females and males differ in the processing of emotional facial expressions including the recognition of emotion, and that emotional facial expressions are detected more rapidly than are neutral expressions. However, whether the sexes differ in the rapid detection of emotional facial expressions remains unclear.

**Methodology/Principal Findings:**

We measured reaction times (RTs) during a visual search task in which 44 females and 46 males detected normal facial expressions of anger and happiness or their anti-expressions within crowds of neutral expressions. Anti-expressions expressed neutral emotions with visual changes quantitatively comparable to normal expressions. We also obtained subjective emotional ratings in response to the facial expression stimuli. RT results showed that both females and males detected normal expressions more rapidly than anti-expressions and normal-angry expressions more rapidly than normal-happy expressions. However, females and males showed different patterns in their subjective ratings in response to the facial expressions. Furthermore, sex differences were found in the relationships between subjective ratings and RTs. High arousal was more strongly associated with rapid detection of facial expressions in females, whereas negatively valenced feelings were more clearly associated with the rapid detection of facial expressions in males.

**Conclusion:**

Our data suggest that females and males differ in their subjective emotional reactions to facial expressions and in the emotional processes that modulate the detection of facial expressions.

## Introduction

Rapid communication via facial expressions is fundamental to human social interaction. The ability to immediately detect emotional signals from facial expressions has been understood as an evolutionary mechanism that enables the receiver to interpret emotional states and to anticipate future actions [1]. Consistent with this notion, several behavioral studies have demonstrated the rapid detection of the emotional facial expressions of others [2], [3].

Given the evidence for sex differences in other cognitive functions, such as verbal fluency and visual perception [4], it has been proposed that females and males differ in their processing of emotional facial expressions as a result of evolution [5], [6]. Consistent with this idea, empirical studies have reported sex differences in the ability to recognize emotion based on facial expressions. For example, several studies have found that females more accurately or sensitively recognized emotional facial expressions than did males [7]–[11]. Meta-analytic studies have also supported a small but significant female advantage in the ability of adults [12] and children and adolescents [13] to recognize the emotions portrayed in facial expressions. However, some studies have reported that females were superior at decoding facial expressions only for some emotional categories [14]–[16]. Other studies have reported no sex differences in the recognition of emotional facial expressions [17], [18]. Taken together, these data appear to suggest sex differences in the recognition of emotional facial expressions, although the patterns characterizing such differences remain inconclusive.

Sex differences in the subjective emotional reactions to emotional stimuli have also been demonstrated. Indeed, a previous study found sex differences in the subjective emotional ratings of emotional facial expressions [19]. Such data are consistent with another line of evidence regarding sex differences in emotional experience using non-facial stimuli, including scenes [20] and autobiographical memories [21], [22].

In contrast to studies showing sex differences in the processing of emotional facial expressions, findings on the rapid processing of emotional facial expressions has been unclear with regard to sex differences. The rapid detection of emotional facial expressions is a critical component of the processing of facial expressions and allows immediate responses to others or the environment [23]. Several previous experimental studies using the visual search paradigm to investigate this issue have demonstrated that the reaction time (RT) for detecting an emotional face (e.g., angry, happy) was shorter than was that for detecting a neutral face [2], [3], and such rapid detection was attributed to the emotional significance of the facial stimuli rather than to their visual features [3]. However, these studies did not examine sex differences. Indeed, few studies have investigated sex differences in the detection of emotional facial expressions [24], [25]. These studies examined the detection of emotional facial expressions among crowds of neutral facial expressions but have reported inconsistent findings. One study reported that males detected angry expressions more rapidly than did females [24], and the other study reported no sex differences in the detection of emotional facial expressions [25]. Thus, it remains difficult to reach conclusions about sex differences in the detection of emotional facial expressions based on these findings. Additionally, these studies focused only on differences in the detection of emotional facial expressions (e.g., anger vs. fear or anger vs. happiness) and did not compare the detection of emotional versus emotionally neutral facial expressions or consider the effect of visual factors. The possibility that females and males differ in the efficient detection of emotional versus neutral facial expressions under conditions in which visual features are controlled remains unexamined.

Furthermore, no study has assessed sex differences in the relationship between subjective emotional experience and the detection of emotion in response to emotional facial expressions. Several neurocognitive models have proposed that the efficient detection of emotional compared with neutral facial expressions may be accomplished through the process involved in detection of the emotional significance of facial expressions, and that emotional processing then modulates the visual processing of the facial expressions [26]–[28]. Consistent with this notion, a previous study using the visual search paradigm showed that higher levels of arousal were related to faster detection of emotional facial expressions [3]. These studies indicate that the efficient attentional capture by emotional, relative to neutral, faces may enhance subjective awareness. However, the possibility that females and males differ with regard to the relationship between subjective emotional ratings and the detection of emotion in response to emotional facial expressions remains untested.

In the present study, we investigated sex differences in the detection of emotional facial expressions using the visual search paradigm. We used facial expressions depicting anger and happiness as target stimuli presented within crowds of neutral expressions according to a previous study [25]. We also presented their anti-expressions as targets following a previous study [3]. Anti-expressions were created by using a morphing technique that produced changes that were equivalent to those produced in the normal emotional facial expressions compared with neutral expressions, but the anti-expressions were usually recognized as emotionally neutral [29]. This method allowed us to determine whether the sex differences in detection performance were attributable to basic visual processing or to emotional significance. To investigate the emotional processes related to facial expression detection, we required participants to rate the subjectively experienced arousal and valence [30]. We also tested stimulus familiarity and naturalness as possible confounding factors [31]. Previous studies showing a female advantage in the recognition of emotional facial expressions led to the expectation of sex differences in the detection or subjective ratings of emotional facial expressions. However, as mentioned above, evidence regarding sex differences in the processing of emotional facial expressions is not consistent, and data directly relevant to the present study are scarce. Therefore, we conducted an exploratory investigation of whether females and males could differ in the RTs for detecting normal versus anti-expressions. Furthermore, we investigated sex differences in subjective emotional experiences of arousal and valence and in the relationship between emotional experiences and detection performance in response to facial expressions.

## Materials and Methods

### Ethics Statement

This study was approved by the local ethics committee of Primate Research Institute, Kyoto University. Written informed consent was obtained from all participants.

### Participants

Ninety volunteers (44 females and 46 males) participated in this study. Females (*M* ± *SD* age, 23.3±5.3) and males (*M* ± *SD* age, 22.5±3.4) did not differ with regard to age, *t*(88) = .8, *p* = .4. All participants were right-handed as assessed by the Edinburgh Handedness Inventory [32] and had normal or corrected-to-normal visual acuity.

### Stimuli

**Figure 1:**
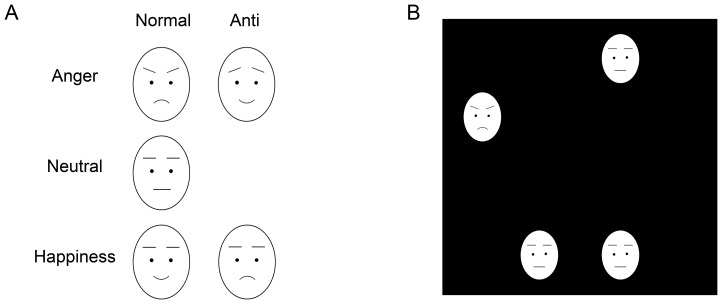
Schematic illustrations of stimuli (A) and visual search display (B). Actual stimuli were photographic faces (see Figure 1 in [3]).

Normal and anti-expressions of angry and happy faces were used as target stimuli, and neutral expressions were used as distractor stimuli. The stimuli were identical to those used in a previous study [3]. Each individual face subtended a visual angle of 1.8° horizontally ×2.5° vertically. The schematic illustrations of stimuli are shown in Figure 1A.

Normal expressions were gray-scale photographs depicting angry, happy, and neutral expressions of a female (PF) and male (PE) model chosen from a facial expression database [33]. Neither model was familiar to any of the participants. No expression showed bared teeth.

Anti-expressions were created from these photographs using computer-morphing software (FUTON System, ATR) on a Linux computer. First, the coordinates of 79 facial feature points were identified manually and realigned based on the coordinates of the bilateral irises. Next, the differences between the feature points of the emotional (angry and happy) and neutral facial expressions were calculated. Then, the positions of the feature points for the anti-expressions were determined by moving each point by the same distance in the direction opposite from that in the emotional faces. Minor color adjustments by a few pixels were performed using Photoshop 5.0 (Adobe).

Two types of adjustments were made to the stimuli using Photoshop 5.0. First, the photographs were cropped into a circle, slightly inside the frame of the face, to eliminate contours and hairstyles not relevant to the expression. Second, the photographs were prepared so that significant differences in contrast were eliminated, thereby removing possible identifying information.

We prepared eight positions, separated by 45 degrees and arranged in a circle (10.0°×10.0°), for the presentation of stimulus faces. Stimuli occupied four of the eight positions; half were presented to the left and half were presented to the right side. A schematic illustration of an example of a stimulus display is presented in Figure 1B. Each combination of the four positions was presented an equal number of times. In the target-present trials, the position of the target stimulus was randomly chosen; however, the target stimulus was presented to the left side in the half of the trials and to the right side in the other half of the trials. In the target-absent trials, all four faces were identical and depicted neutral expressions.

### Procedure

Stimulus presentation was controlled by Presentation 14.9 (Neurobehavioral Systems) and was implemented on a Windows computer (HP Z200 SFF, Hewlett-Packard Company). The stimuli were presented on a 19-inch CRT monitor (HM903D-A, Iiyama) with a refresh rate of 150 Hz and a resolution of 1024×768 pixels. The refresh rate was confirmed by using a high-speed camera (EXILIM FH100, Casio) with a temporal resolution of 1000 frames/s.

The experiment was conducted in an electrically shielded and soundproofed room (Science Cabin, Takahashi Kensetsu). Participants sat in chairs with their chins fixed into steady positions 80 cm from the monitor. They were asked to keep their gaze on the fixation cross (0.9°×0.9°) at the center of the display when the cross was presented. Before the experiment began, participants engaged in 20 practice trials to gain familiarity with the apparatus.

The experiment consisted of a total of 432 trials presented in six blocks of 72 trials, with an equal number of target-present and target-absent trials. The trial order was pseudo-randomized. In each trial, the fixation cross was presented for 500 ms and then the stimulus array consisting of four faces was presented until participants responded. Participants were asked to respond as quickly and accurately as possible by pushing the appropriate button on a response box (RB-530, Cedrus) using their left or right index finger to indicate whether all four faces were the same or one face was different. The position of the response buttons was counterbalanced across participants.

After the visual search task, participants engaged in the rating task for the target and distractor (neutral) facial stimuli. The stimuli were presented individually. They were asked to evaluate each stimulus in terms of emotional arousal and valence (i.e., the intensity and nature of the emotion, respectively, that participants felt when perceiving the stimulus expression) [30] using a nine-point scale ranging from 1 (low arousal and negative, respectively) to 9 (high arousal and positive, respectively). They were also asked to rate familiarity (i.e., the frequency with which they encountered facial expressions such as those depicted by the stimulus in daily life) and naturalness (i.e., the degree to which the expression depicted by the stimulus seemed natural) using a nine-point scale ranging from 1 (not at all) to 9 (very much) to test possible confounding factors [31]. The order of facial stimuli and rating items during the rating task were randomized and balanced across participants.

### Data analysis

All statistical tests for the behavioral data were performed using the SPSS 10.0J software (SPSS Japan), and statistical significance was set at *p*<.05.

*RT*. The mean RTs of correct responses in target trials were calculated for each condition, excluding measurements ±3 *SD* from the mean as artifacts. The RTs were then subjected to a three-way repeated-measure ANOVA with type (normal/anti-expression) and emotion (anger/happiness) as within-participant factors, and sex (female/male) as a between-participant factor. Follow-up analyses of significant interactions for the simple effect were conducted [34]. When higher-order interactions were significant, the main effects or lower-order interactions were not subjected to interpretation because they would be qualified by higher-order interactions [35].

Preliminary analyses showed that accuracy was high under all conditions (*M* ± *SE* %; normal-anger: 91.8±1.4, 91.9±1.6; normal-happy: 92.2±1.2, 91.7±1.4; anti-anger: 80.8±2.2, 78.9±2.5; anti-happy: 81.8±2.3, 80.6±2.4 for females and males, respectively), and we found no evidence of a speed–accuracy tradeoff. Therefore, we report only the RT results.

#### Rating

Each rating of arousal, valence, familiarity, and naturalness was analyzed according to the protocol used for the RT analysis (i.e., ANOVA and follow-up analyses).

#### Relationship between ratings and RTs

Multiple regression analyses were performed to examine the relationship between subjective ratings and RTs. First, separate analyses were conducted for females and males. In these analyses, the mean RT for each participant under each condition (normal-anger, normal-happiness, anti-anger, and anti-happiness) was used as the dependent variable to test the between-response variability (vs. the between-participant variability) [36]. The independent variable was the rating for arousal, valence, familiarity, or naturalness (effect of interest), and dummy variables were used to represent participants (effects of no-interest).

To examine sex differences in the relationship between ratings and RTs, we then tested for differences in the slopes of the regression lines for females and males. The independent variables were the interaction between sex and rating (effect of interest) and the main effects of sex and participant (effects of no-interest). Adjusted RTs were calculated to plot the relationship between rating and RTs by partialling out the group mean and the effect of participant.

## Results

### RT

**Figure 2:**
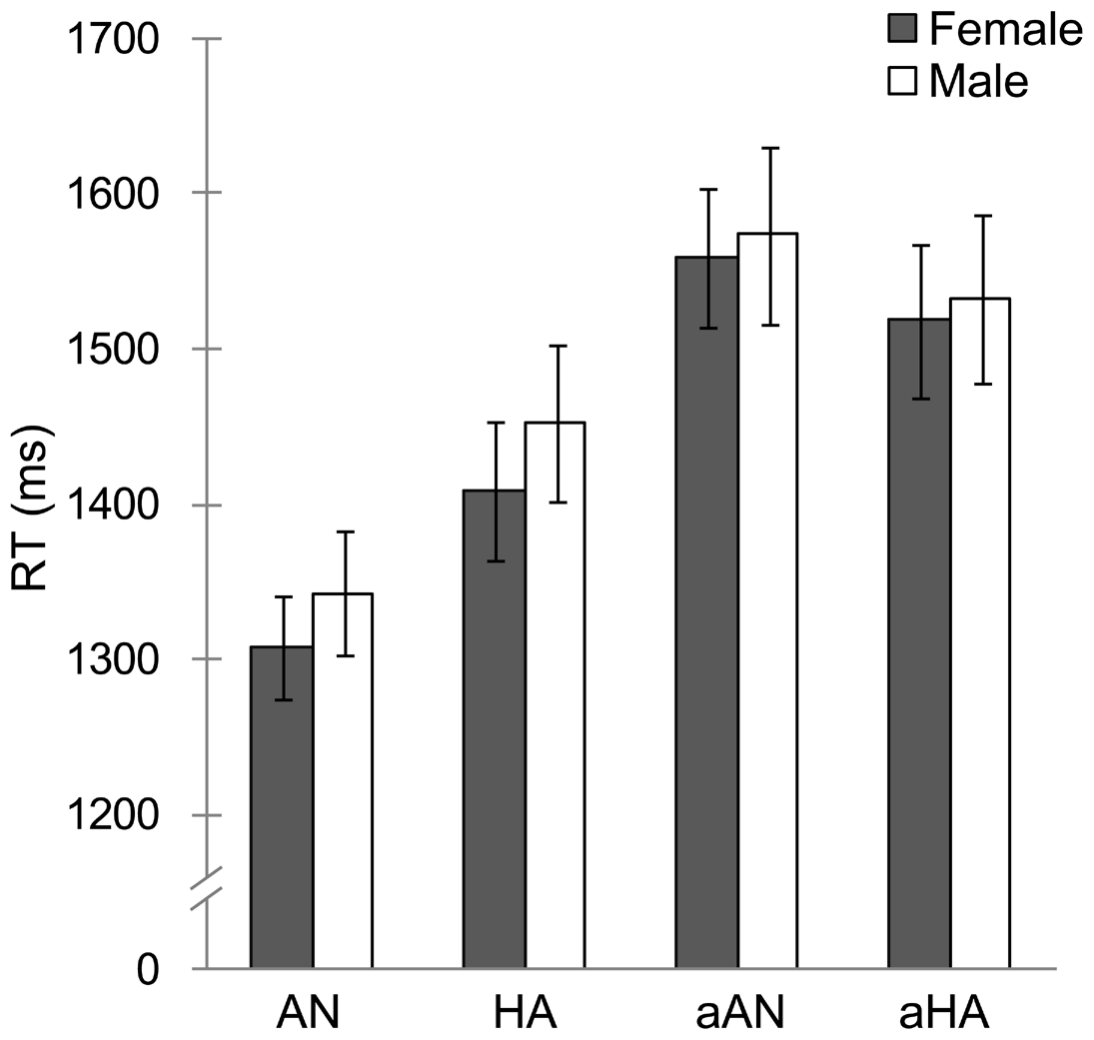
Mean (with SE) reaction time (RT) for each target stimulus condition. AN  =  normal-anger; HA  =  normal-happiness; aAN  =  anti-anger; aHA  =  anti-happiness.

In terms of RTs (Figure 2), the three-way ANOVA with type, emotion, and sex as factors revealed significant main effects of type, *F*(1, 88) = 92.7, *p*<.001, and emotion, *F*(1, 88) = 6.8, *p*<.05, as well as a significant interaction between type and emotion, *F*(1, 88) = 21.8, *p*<.001. No other main effects or interactions were significant, *F*(1, 88)<.5, *p*>.1, indicating no significant sex differences in the RTs to facial targets.

To assess the general patterns of RTs across females and males, follow-up analyses were conducted for the interaction of type × emotion. The results revealed that the simple effects of type were significant for both anger, *F*(1, 176) = 105.8, *p*<.001, and happiness, *F*(1, 176) = 16.5, *p*<.001, indicating shorter RTs for the normal expressions of both anger and happiness than for their anti-expressions. The simple effect of emotion was significant for normal expressions, *F*(1, 176) = 27.8, *p*<.001, indicating shorter RTs for normal-anger than for normal-happiness, but not for anti-expressions, *F*(1, 176) = 3.2, *p* = .08.

### Ratings

**Figure 3:**
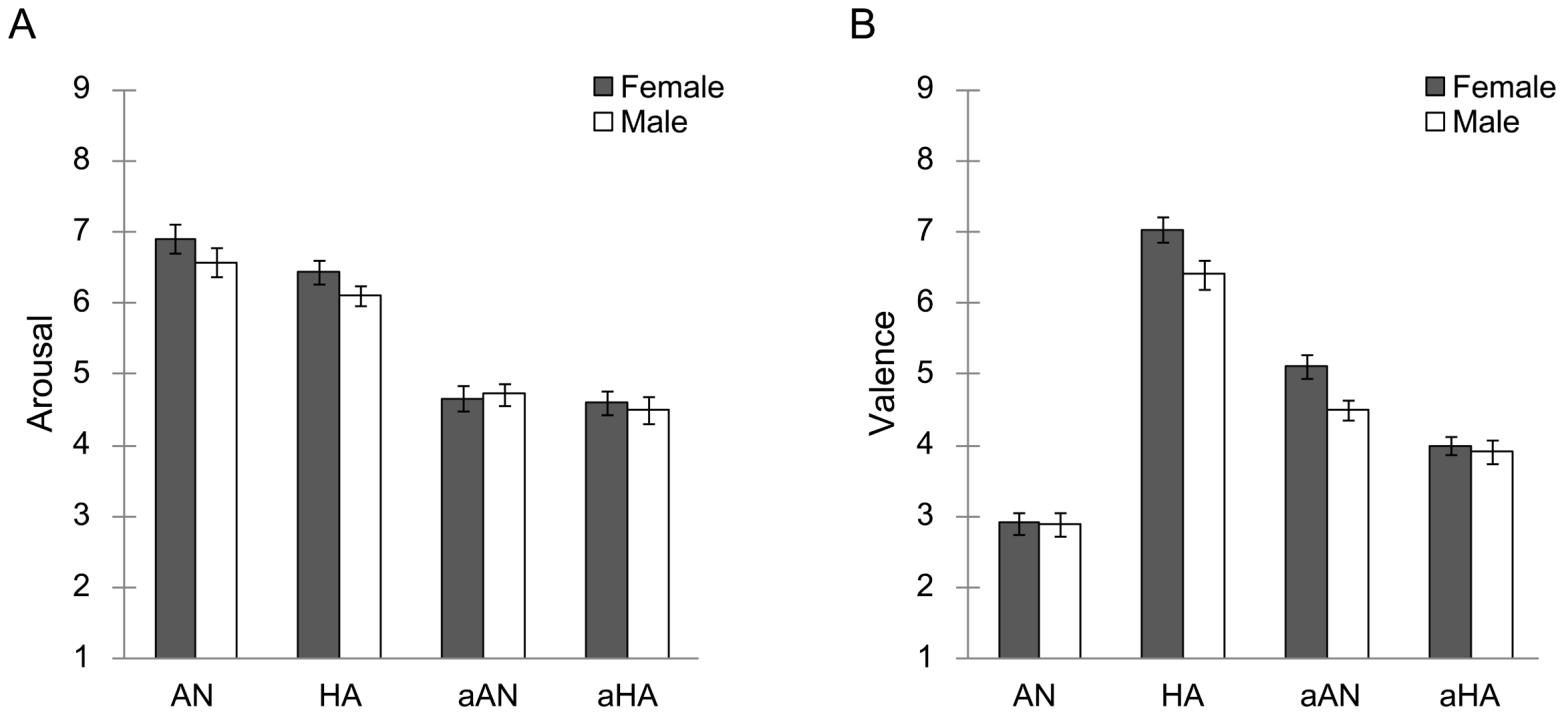
Mean (with SE) ratings of arousal (A) and valence (B) for each target facial expression. AN  =  normal-anger; HA  =  normal-happiness; aAN  =  anti-anger; aHA  =  anti-happiness.

In terms of arousal ratings (Figure 3A), the three-way ANOVA with type, emotion, and sex as factors revealed a significant main effect of type, *F*(1, 88) = 218.1, *p*<.001, indicating higher arousal to normal expressions than for anti-expressions, and of emotion *F*(1, 88) = 10.1, *p*<.005, indicating higher arousal for angry than for happy expressions. No other main effects and interactions were significant, *F*(1, 88)<1.5, *p*>.1, indicating no significant sex differences in evaluations of emotional arousal.

In terms of valence ratings (Figure 3B), the three-way ANOVA revealed significant main effects of type, *F*(1, 88) = 17.7, *p*<.001; emotion, *F*(1, 88) = 233.0, *p*<.001; and sex, *F*(1, 88) = 7.1, *p*<.01. We also observed a significant two-way interaction between type and emotion, *F*(1, 88) = 332.0, *p*<.001, and a significant three-way interaction among type, emotion, and sex, *F*(1, 88) = 4.9, *p*<.05. No other interactions were significant, *F*(1, 88)<06, *p*>.1. Follow-up analyses for the three-way interaction revealed a significant simple effect of sex for normal-happy, *F*(1, 352) = 7.6, *p*<.01, and for anti-anger, *F*(1, 352) = 7.2, *p*<.01, expressions, indicating that females experienced more pleasant emotions in response to normal-happy and anti-angry expressions than did males.

With regard to familiarity ratings (Figure S1A), the three-way ANOVA revealed significant main effects of type, *F*(1, 88) = 51.3, *p*<.001, emotion, *F*(1, 88) = 157.7, *p*<.001, and sex, *F*(1, 88) = 4.7, *p*<.05, and a significant interaction between type and emotion, *F*(1, 88) = 240.4, *p*<.001. We observed no other significant interactions, *F*(1, 88)<1.4, *p*>.1. The main effect of sex indicated that females experienced the target stimuli as more familiar facial expressions than did males.

For naturalness ratings (Figure S1B), the three-way ANOVA revealed a significant main effects of type, *F*(1, 88) = 19.3, *p*<.001; and emotion, *F*(1, 88) = 82.5, *p*<.001, and a significant two-way interaction between type and emotion, *F*(1, 88) = 137.8, *p*<.001. No other significant main effect and interactions, *F*(1, 88)<1.0, *p*>.1, indicating no sex differences in evaluations of naturalness.

### Relationship between ratings and RTs

**Figure 4:**
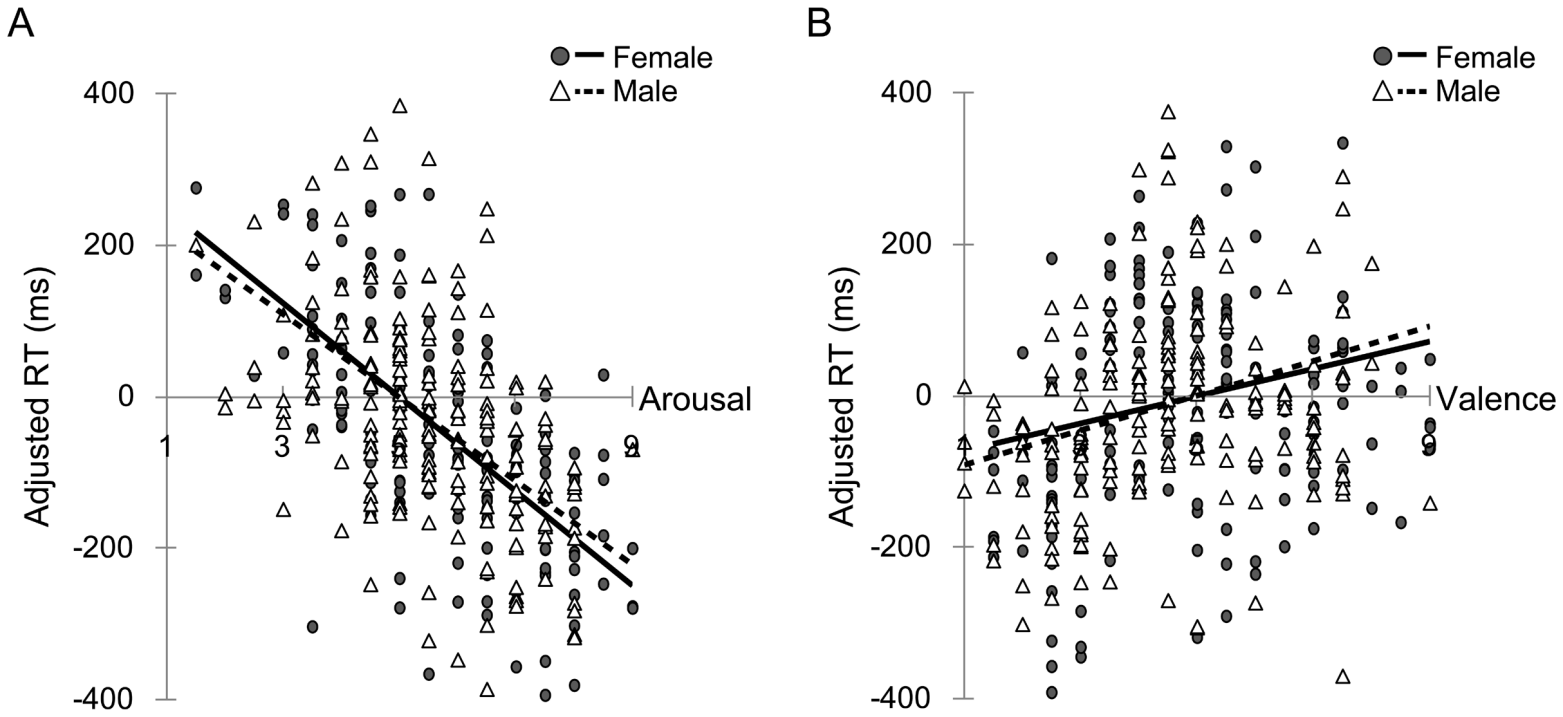
Sex differences in the relationship between arousal and RT (A) and between valence and RT (B). The scatter plots and regression lines indicate relationships between ratings and adjusted RTs.

With respect to the relationship between arousal ratings and RTs (Figure 4A), we first conducted separate multiple regression analyses for females and males. The results showed that the negative relationship between arousal ratings and RTs was significant in both females, *t*(131) = −6.9, *p*<.001, and males, *t*(137) = −5.5, *p*<.001, indicating that higher arousal ratings were related to shorter RTs for detecting facial expressions in both sexes. We next tested for sex differences in the relationship between arousal ratings and RTs. The results revealed a significant sex difference, *F*(2, 268) = 38.5, *p*<.001, indicating that females showed a more robust negative arousal–RT relationship than did males.

In terms of the relationship between valence and RTs (Figure 4B), the multiple regression analysis for each sex showed the significant positive valence–RT relationship in both females, *t*(131) = 2.2, *p*<.05, and males, *t*(137) = 2.7, *p*<.01, indicating that more negative feelings were related to shorter RTs for detecting target facial expressions in both females and males. The test for differences in the slopes revealed the significant sex difference in the valence–RT relationship, *F*(2, 268) = 6.0, *p*<.01, indicating a stronger positive valence–RT relationship in males than in females.

No significant relationship was found between familiarity and RT in females, *t*(131) = .4, *p* = .7, or males, *t*(137) = 1.5, *p* = .1, or between naturalness and RT in females, *t*(131) = 1.0, *p* = .3, or males, *t*(137) = .4, *p* = .7. The test for differences in the slopes also revealed no sex difference in the relationship between familiarity and RT, *F*(2, 268) = 1.2, *p* = .3, or between naturalness and RT, *F*(2, 268) = .6, *p* = .6.

## Discussion

The general patterns of RTs across females and males showed that normal expressions of both anger and happiness were detected more rapidly than were their anti-expressions and that normal-anger expressions were detected more rapidly than were normal-happy expressions. The subjective ratings revealed that normal expressions elicited more arousal than did anti-expressions, and that normal-angry expressions elicited more negatively valenced emotion than did normal-happy expressions. Moreover, regression analyses showed a significant negative relationship between arousal and RTs, indicating that higher levels of emotional arousal facilitated rapid detection of facial expressions. Collectively, these results are consistent with those of a previous study [3]. However, the regression analyses in this study also revealed a positive relationship between valence and RTs, indicating that more unpleasant feelings were associated with more rapid detection of facial expressions. This discrepancy is attributable to the superior statistical power of the large sample used here compared with that in the previous study (*n* = 90 vs. 17). Taken together, RT and rating data indicate that humans, regardless of sex, detect emotional facial expressions more rapidly than anti-expressions, that they detect normal-angry expressions more rapidly than normal-happy expressions, and that such rapid detection of facial expressions is related to emotional elicitation.

More important, the current study investigated sex differences in the rapid detection of emotional facial expressions and the relationship between such differences and subjective emotion. The RTs showed that females and males performed equally in tasks involving the rapid detection of emotional versus neutral and angry versus happy facial expressions. This result is consistent with those of one previous visual search study [25] that found no sex difference in the detection of emotional facial expressions of anger and happiness, but it is not consistent with those of another previous study [24] that reported male superiority in detecting angry expressions. However, these previous studies did not compare the detection of emotional versus emotionally neutral facial targets. This is the first study to show the absence of sex differences in the rapid detection of emotional versus neutral facial expressions. Furthermore, because the anti-expressions contained changes in visual features comparable to those in emotional facial expressions [29], our results regarding emotional versus neutral expressions cannot be attributable to basic visual processes. Additionally, the difference in detection performance in response to angry versus happy facial expressions is difficult to explain in terms of visual factors because comparable detection results were obtained for their anti-expressions. In summary, our data suggest that females and males are equally efficient at detecting emotional versus neutral and angry versus happy expressions.

In contrast, we found sex differences in the subjective emotional ratings offered in response to the facial expressions. Specifically, females accorded higher valence ratings to normal-happy and anti-angry expressions than did males. Because valence reflects the quality of an emotional experience [30], these results suggest that females feel more qualitatively in response to others' emotional facial expressions than do males. These results are consistent with those of several previous studies showing that females were highly sensitive to their emotional experiences [20]–[22]. The results are also in line with a previous report that females, compared with males, recognize more extreme emotions in emotional facial expressions [7], [9]. Our results suggest that females and males differ in their emotional reactions to facial expressions.

Furthermore, the results of regression analyses revealed sex differences in the relationship between subjective ratings and RTs. The relationship between arousal and RTs, in which more arousing expressions were more rapidly detected, was more evident among females than among males. In contrast, the relationship between valence and RTs, in which more negatively experienced expressions were more rapidly detected, was more evident among males than among females. Taken together, these results indicate that females and males differ in their emotional reactions to others' facial expressions and that these differing reactions modulate the detection of facial expressions in different ways. Several neurocognitive models have proposed that the efficient detection of emotional facial expressions may initially involve processing the emotional significance of facial expressions in subcortical regions such as the amygdala [26]–[28]. These models postulate that the result of such emotional processing then modulates the visual processing of the facial expressions that occurs in the cortical visual areas. Consistent with this assumption and our results, several neuroimaging studies have shown that females and males showed different patterns of amygdala activation in response to emotional facial expressions [37], [38]. However, no study has shown the functional significance of such sex differences in amygdala activation with regard to emotional facial expressions. The present study is of great significance as we believe this is the first report of the effect of sex differences on the emotional modulation involved in the detection of emotional facial expressions. Our findings implicate sex differences in the neural mechanisms involved in the rapid detection of emotional facial expressions.

The sex difference in the relationship between subjective emotional ratings and the detection of emotional facial expressions appears to be consistent with evolutionary evidence regarding sex differences. Females have been more responsible for childrearing than have males, and it has been hypothesized that females show greater sensitivity to emotionally arousing facial expressions across-the-board, as a result of their evolutionary role as primary caretakers because mothers must rapidly respond to the emotional signals of their infants to increase the chances of the infants' survival [5]. Our results showing sex differences in the psychological processes underlying the rapid detection of emotional facial expressions suggest that the enhanced emotional arousal demonstrated by females expedites their efficient detection of the important signals communicated by emotional facial expressions. In childrearing situations, these expressions are often related to the status of infants, and the ability of females to rapidly detect them helps to maintain infant health and produce prosocial outcomes. This quantitative modulation of rapid detection of emotional facial expressions by subjective emotional processing in females may be also consistent with the general female advantage in processing emotional facial expressions, which has been shown in previous empirical studies [7]–[11], [6]. In contrast, from an evolutionary perspective, males are more likely than females to have been subjected to aggressive behavior from other males in the context of mating or hunting and such situations can result in death or serious injury [39], [40]. In this context, some researchers have shown that males generally express and endorse emotions through their actions including aggressive behaviors [41]. Based on this literature, our results suggest that, among males, subjective negative feelings would accelerate the efficient detection of the aggressive signals communicated by others via emotional facial expressions. This qualitative modulation of the detection of emotional facial expressions by subjective emotional processing is consistent with a previous study showing a male advantage in the rapid detection of angry faces [24] and may account for discrepancies between our RT result and the female advantage in emotional processing noted by previous studies [7]–[11]. Taken together, our findings suggest that differences in the evolutionary roles or traits of females and males may have led to the development of sex differences in the psychological processes underpinning the rapid detection of emotional facial expressions.

Irrespective of these possible evolutionary interpretations, it must be noted that learned factors may also account for the results. Some researchers have reported that social factors may contribute to sex differences in the psychological processes underlying the rapid detection of emotional facial expressions. For example, it has been shown that the intensity of reported emotions is correlated with belief in the stereotypical social role of females and males [42]. Specifically, females who believed more strongly in stereotypical role patterns reported more intense emotions in response to emotional scenes, and males who believed more strongly in stereotypical role patterns reported less intense emotions in response to such scenes. These data suggest that social factors, such as gender role stereotypes, modulate the relationship between subjective emotional feelings and the rapid detection of emotional facial expressions.

Our results showed that detection RT was not related to ratings of the familiarity or naturalness of facial targets, suggesting that these non-emotional processes cannot account for detection performance. However, irrespective of relationships involving facial expression detection, females reported more familiarity with the stimulus facial expressions than did males. This result suggests that females have a better memory for the various types of facial expressions that they observe in their daily lives, which is consistent with a previous study reporting that females retained a better memory for emotional events than did males [22]. These data suggest that consideration of sex differences in memory for emotional facial expressions may be a promising topic for future investigation.

Several limitations of the present study should be noted. First, we used stimulus faces of only one female and one male model from standardized materials of facial expressions [33] because a computer-morphing technique by which the anti-expressions were created can be applied only to faces with closed mouths [29]. This approach may have confounded the effect of sex with that of the identity of facial stimuli. Some previous studies have shown that the sex of the target stimuli affects the emotional processing of facial expressions, in that angry expressions depicted by male faces are recognized more accurately or more rapidly, whereas happy expressions depicted by female faces are recognized more accurately and more rapidly [25], [43], [44]. Therefore, further investigations of the effect of the sex of target stimuli are warranted.

Second, we used only two emotional facial expressions, anger and happiness. Our primary purpose was to investigate sex differences in the detection of emotional compared with emotionally neutral facial expressions. For this purpose, target stimuli with both negative (anger) and positive (happy) affects might be effective. However, some researchers have found sex differences in the recognition of facial expressions depicting certain categories of emotion [14]–[16]. Therefore, further investigations using more categories of emotion are needed to investigate the effects of different emotional information on sex differences in detecting facial expressions.

In summary, our results showed no sex differences in the rapid detection of emotional compared with emotionally neutral expressions. However, we did observe sex differences in the subjective ratings of facial stimuli and the relationship between ratings and RTs. Females reported a stronger qualitative response to the emotional facial expressions of others than did males. Furthermore, emotional arousal enhanced the detection of facial expressions more strongly in females than in males, whereas negative feelings facilitated the detection of facial expressions more clearly in males than in females. These findings suggest females and males differ in their subjective emotional reactions to facial expressions and that this difference leads to subsequent differences in the ways in which emotion modulates the detection of emotional facial expressions.

## Supporting Information

Figure S1 **Mean (with** ***SE*** **) ratings of familiarity (A) and naturalness (B) for each target facial expression.** AN  =  normal-anger; HA  =  normal-happiness; aAN  =  anti-anger; aHA  =  anti-happiness.
(TIF)
