# Supplementary figures and images for: Sex Differences in the Rapid Detection of Emotional Facial Expressions
Source: PLoS One. 2014 Apr 11;9(4):e94747. doi: 10.1371/journal.pone.0094747 (PMC3984253; doi:10.1371/journal.pone.0094747)

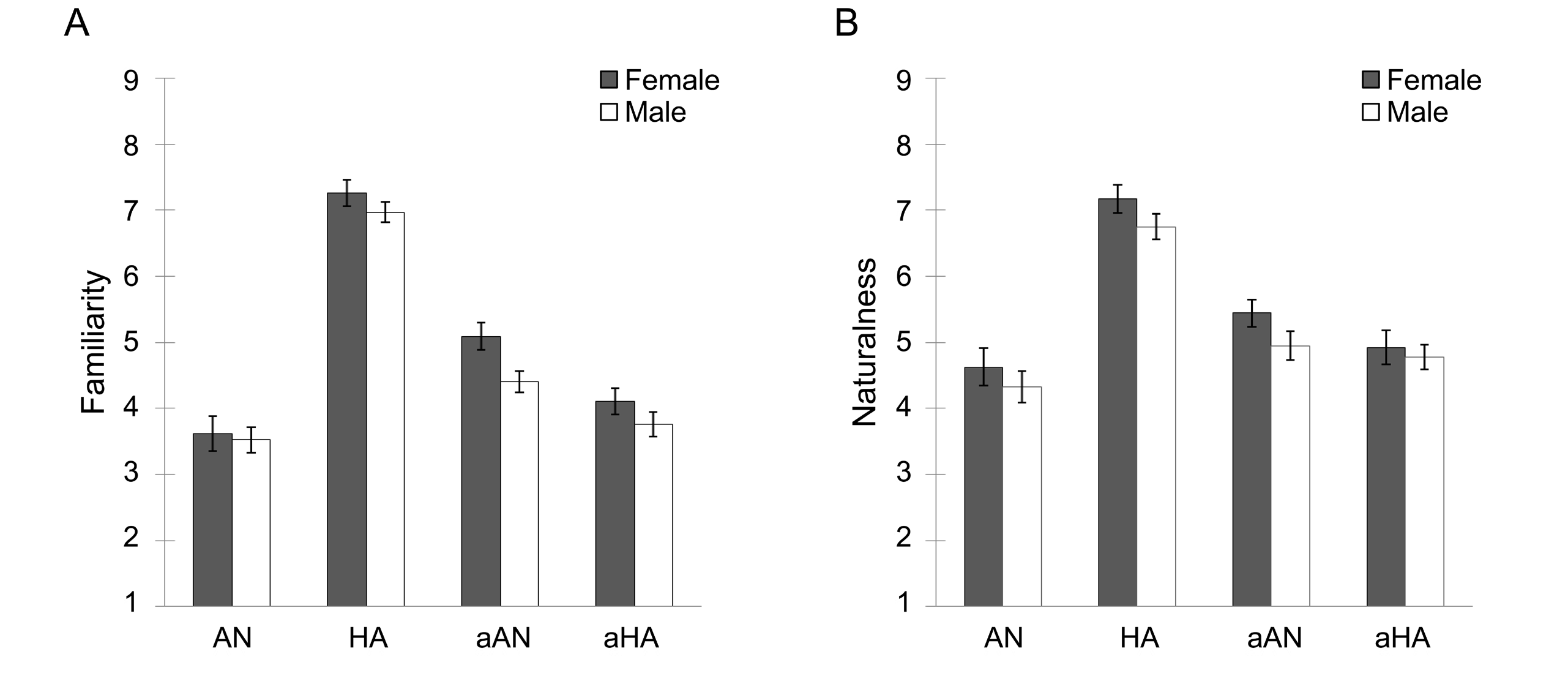

Supplement: Figure S1 — Mean (with SE ) ratings of familiarity (A) and naturalness (B) for each target facial expression. AN = normal-anger; HA = normal-happiness; aAN = anti-anger; aHA = anti-happiness. (TIF) [file pone.0094747.s001.tif]
